# Surface Changes Induced by Brushing Increase *Candida albicans* Biofilms on 3D-Printed Denture Base Resin

**DOI:** 10.3390/jof11090668

**Published:** 2025-09-12

**Authors:** Rafaelly Camargo, Jonatas Silva de Oliveira, Amanda Costa Ferro, Beatriz Ribeiro Ribas, Alan Augusto Valério Alves, Janaina Habib Jorge

**Affiliations:** Department of Dental Materials and Prosthodontics, School of Dentistry, São Paulo State University (UNESP), Araraquara 14801-903, São Paulo, Brazil; rafaelly.camargo@unesp.br (R.C.); jonatas.oliveira@unesp.br (J.S.d.O.); a.ferro@unesp.br (A.C.F.); beatriz.ribas@unesp.br (B.R.R.); alan.augusto@unesp.br (A.A.V.A.)

**Keywords:** denture base, 3D printing, brushing, roughness, *Candida albicans*

## Abstract

Studies assessing the long-term effects of brushing with cleaning and disinfecting agents on surface roughness and biofilm accumulation on the three-dimensionally (3D) printed resins remain scarce. This study aimed to evaluate the effects of different solutions and simulated brushing times on the surface roughness and biofilm formation on heat-cured and 3D-printed denture base resins. Discs samples (10 mm × 1.2 mm) were prepared and randomly assigned to brushing treatments using the following solutions: distilled water, disinfectant liquid soap and dentifrice. The specimens (n = 9) were subjected to 10,000, 20,000 and 50,000 cycles to simulate 1, 2 and 5 years of brushing, respectively. The control group consisted of unbrushed samples. Surface roughness (Ra) was measured. *Candida albicans* biofilm formation was evaluated by counting colony forming units, cellular metabolic activity, and qualitative and quantitative analysis through confocal fluorescence microscopy. A significant increase in roughness was observed in both resins after two years of simulated brushing, mainly with dentifrice. After two years of brushing, an increase in the number of cells and metabolism of *C. albicans* was observed, in agreement with the fluorescence and biofilm thickness results. Brushing with dentifrice increased the roughness of heat-cured and 3D-printed resins and potentially increased *C. albicans* biofilm formation.

## 1. Introduction

Dental prostheses are traditionally made from heat-activated acrylic resins based on polymethyl methacrylate (PMMA), which is a material that possesses adequate mechanical properties, low cytotoxicity, and simple processing [1,2,3]. However, the prolonged time required to manufacture prostheses, changes in physical and mechanical properties and the release of residual monomers, potentially causing allergic reactions, constitute relevant limitations to their use [4,5]. Alternatively, CAD/CAM (Computer-Aided Design/Computer-Aided Manufacturing) technology has been widely used in the manufacture of dental prostheses through subtractive (milling) or additive (three-dimensional printing) manufacturing techniques [6].

Additive manufacturing, or 3D printing, is a rapid prototyping technology that allows the construction of three-dimensional objects under computational control, in which materials are combined or solidified together (such as liquid molecules or powder grains) based on a predefined design in software [7]. Among the available methods, stereolithography (SLA) and digital light processing (DLP), differing mainly in the light source [8,9]. In DLP, the liquid photopolymer is exposed to flashes of ultraviolet light digitally projected onto the printed area, curing one layer of resin at a time, producing highly accurate objects [8]. Compared to heat activated and milled resins, 3D printing has a lower cost, greater material utilization and the possibility of producing multiple prostheses simultaneously [10]. Furthermore, this technology designed for dental applications provides more personalized, simplified and efficient patient care [7].

Regardless of the type of resin [11,12], oral dentures are subject to microbial adhesion and biofilm accumulation, especially at the interface between the internal surface of the denture and the oral mucosa, which constitutes a unique ecological niche for the colonization of microorganisms, due to the relatively anaerobic environment with reduced pH [13,14]. Therefore, daily cleaning of dentures is essential and should be done using a combination of chemical methods (immersion in disinfectant solutions) and mechanical methods (brushing with neutral soap or toothpaste) to prevent microbial proliferation and invasion of oral tissues [15,16,17].

Brushing with soft-bristled toothbrushes and neutral soap is recommended, but the use of toothpaste is more common due to its practicality, low cost and pleasant taste [18]. However, over time, the friction caused by the toothpaste and the brush can increase the roughness of the internal surface of the denture, favoring microbial adhesion, especially of *Candida albicans* [19,20]. Lifebuoy^®^ soap has been studied as an alternative disinfectant solution for soaking and brushing dentures and, in short-term evaluations, did not cause adverse effects on acrylic resins [21,22,23].

Although several studies have investigated the effects of different hygiene protocols on the surface and mechanical properties of acrylic resins, as well as those affecting microorganisms, it is still necessary to evaluate the long-term impacts of these practices to ensure satisfactory prosthetic results and the reduction of oral diseases associated with the use of dental prostheses, such as denture stomatitis [15,21,24,25]. Furthermore, despite the increasing use of three-dimensionally printed resins in the manufacture of dental prostheses, there is still a lack of studies investigating the effects of brushing associated with cleaning and disinfection agents on the roughness and biofilm formation by *C. albicans* on these resins.

This study is justified by the need to add information on the impacts of prolonged mechanical brushing with toothpaste and disinfectant soap on printed resins. The objective of this study was to analyze the effects of simulated mechanical brushing, in different periods, on the roughness and biofilm formation on denture base resins obtained by the conventional method and by 3D printing. The null hypotheses tested were: (1) simulated brushing with toothpaste and disinfectant soap for 1, 2 and 5 years does not alter the roughness of conventional, and 3D-printed resins; and (2) simulated brushing during these same periods and conditions does not interfere with the formation, metabolic activity and viability of the *C. albicans* biofilm on the resins.

## 2. Materials and Methods

### 2.1. Sample Preparation

The samples of heat activated acrylic resin (Vipi Wave, Vipi Produtos Odontológicos, Pirassununga, São Paulo, Brazil) were made from metal molds with 10 mm in diameter and 1.2 mm in thickness, according to the manufacturer’s instructions. The manipulated resin was placed between two glass plates previously sandblasted with aluminum oxide to standardize the surface roughness of the samples at approximately 3.0 µm, simulating the internal surface of the dentures [26].

The fabrication of 3D-printed resin samples was based on the protocol described by Shim et al. (2020) [27]. Circular samples, with dimensions of 10 mm in diameter and 1.2 mm in thickness, were virtually designed using CAD software (MeshMixer v. 3. 5. 474, Autodesk Inc., Mill Valley, CA, USA). Printing was performed using 3D FlashForge Hunter (Done 3D Store, Ribeirão Preto, São Paulo, Brazil) and a commercially available composite for making denture bases (Cosmos Denture, Yller Biomateriais S.A., Pelotas, Rio Grande do Sul, Brazil). The samples were printed in 90° orientation [27], with a layer thickness of 50 µm. Post-polymerized and removal of impurities and residual monomer were made according to the manufacturer’s instructions. To simulate the internal surface of dentures, samples were standardized to approximate roughness of 3.0 µm by sanding them with 150-grit silicon carbide sandpaper under running water [28].

Immediately after preparation, all samples were washed in an ultrasonic bath using distilled water for 15 min to remove residues from the preparation process. The samples were then stored in 20 mL of distilled water for 48 h to release residual monomer [29].

### 2.2. Surface Roughness Before Simulated Brushing (T0)

After 48 h of storage, surface roughness of the samples was measured using a profilometer (Surftest SJ-401, Mitutoyo Sul Americana Ltda, Santo Amaro, São Paulo, Brazil) with an accuracy of 0.01 µm. In each sample, three measurements were performed, and the average was obtained. Only samples with roughness values between 2.7 and 3.7 µm (Ra) were selected for testing, simulating the internal surface of the dentures [26].

### 2.3. Experimental Groups

The samples of both resins were randomly divided into experimental groups according to the simulated brushing cycles (0, 1, 2 and 5 years) and according to the substances used: Distilled water (negative control group): the samples were brushed with distilled water; Disinfectant liquid soap solution (experimental group): the samples were brushed with liquid soap; Dentifrice (experimental group): the samples were brushed with dentifrice.

### 2.4. Brushing Cycles

The brushing cycle was performed using soft-bristled toothbrushes (Sorriso Original, Colgate-Palmolive, São Paulo, Brazil) on a six-point brushing machine, with a constant vertical load of 200 g being applied on each specimen and a linear displacement of 18 mm to each side. The samples were exposed to 10,000, 20,000, and 50,000 cycles to simulate 1, 2, and 5 years of manual brushing, respectively, in independent specimens [30,31]. During brushing, the samples were immersed in 100 mL of each substance, according to the experimental group. Brushes and solutions were changed after each 10,000 cycles [23,30].

Regarding the substances used, a solution with a dentifrice (Colgate TOTAL^®^ Creme, Colgate-Palmolive Company, New York, NY, USA) and distilled water was prepared in a 1:1 ratio [32,33]. Disinfectant liquid soap (Lifebuoy^®^, Unilever, London, UK) was used at a concentration of 0.78%, according to a previous study [21]. Distilled water was used as a negative control.

### 2.5. Surface Roughness After Simulated Brushing (Ra)

After the different brushing times (T1, T2 and T5), the samples from each experimental group were subjected to surface roughness reading using a profilometer (Surftest SJ-401; Mitutoyo Sul Americana Ltda, Santo Amaro, São Paulo, Brazil) with an accuracy of 0.01 μm. In each sample, three measurements were performed, and the average was obtained. The Ra parameter, which represents the arithmetic means of the absolute deviations of the roughness profile, was chosen to allow for direct comparison with findings from other studies [23]. All procedures were performed in triplicate and on 3 separate occasions, totaling 9 samples per experimental group (n = 9).

### 2.6. Biofilm Formation

Before microbiological testing, samples of both resins were disinfected by exposure to ultraviolet light under dry conditions for 20 min per side [1,34]. This procedure was adopted to preserve the acrylic resin’s properties. The evaluation of biofilm formation capacity was performed at T0 (without brushing), T1, T2 and T5 (after simulated brushing cycles) through the following tests: analysis of cell proliferation (counting of colony-forming units), by evaluating cellular metabolic activity (alamarBlue^®^ assay, TermoFisher Scientific, Marietta, OH, USA) and by qualitative and quantitative analysis by confocal fluorescence microscopy (CFM).

#### 2.6.1. Assessment of Cell Proliferation by Counting Colony Forming Units (CFU/mL)

*Candida albicans* cells (ATCC^®^ 90028) were grown on Sabouraud Dextrose Agar (SDA) at 37 °C for 48 h, and colonies were inoculated into 10 mL of yeast nitrogen base (YNB) and incubated aerobically at 37 °C for 16 h. A 0.5 mL aliquot of this culture was transferred to 9.5 mL of fresh YNB and incubated for 8 h. Cell density was measured at 540 nm, followed by centrifugation (10,000 rpm, 5 min), two washes Phosphate-Buffered Saline (PBS), and resuspension in Roswell Park Memorial Institute (RPMI-1640) medium to produce standardized suspensions of 1 × 10^6^ CFU/mL.

After disinfection, the specimens were placed in a 24-well plate with 750 µL of the *Candida albicans* suspension and 750 µL of sterile RPMI-1640, totaling 1500 µL per well. The plates were incubated for 90 min in an orbital shaking incubator at 37 °C and 75 rpm to allow adhesion of fungal cells to the surface of the specimens. Then, the contents of the wells were removed, and the samples were washed twice with 1000 µL of PBS to remove non-adhered cells. Afterward, 1500 µL of fresh RPMI-1640 medium was added to each well and the plates were incubated for 48 h at 37 °C for the development of mature biofilm. The culture medium was partially renewed after 24 h by removing 750 µL from each well and adding the same volume of fresh medium [35].

After 48 h of incubation, the biofilms were carefully detached from the specimens by scraping with the tip of a sterile pipette tip for 1 min [24,26,35]. Then, 100 µL of the suspension obtained was transferred to an Eppendorf tube containing 900 µL of sterile PBS. Four serial dilutions were performed, of which 20 µL of the 10^−3^ and 10^−4^ dilutions were seeded in Petri dishes containing SDA supplemented with 0.1 g/L of chloramphenicol. The plates were incubated at 37 °C for 48 h. After this period, the colonies were counted, and the number of colony-forming units per milliliter (CFU/mL) was calculated using the formula:

CFU/mL = (Number of colonies) × 10^n^/q

In this formula, n is equivalent to the absolute value of the chosen dilution and q is equivalent to the quantity, in mL, of each dilution seeded on the plates. All procedures were performed in triplicate and on 3 separate occasions, totaling 9 samples per experimental group (n = 9).

#### 2.6.2. Assessment of Cellular Metabolic Activity Using the alamarBlue^®^ Assay

First, *C. albicans* biofilm was formed on the samples as described in item 2.6.1. Then, the biofilm was washed twice with 1000 µL of PBS. After washing, 1500 µL of sterile RPMI-1640 was added to each well, followed by the addition of 150 µL of alamarBlue^®^ solution. The plates were then placed in an orbital shaking incubator at 37 °C and 75 rpm for 4 h. After this period, 200 µL from each well were transferred, in triplicate, to a 96-well plate, to perform fluorescence measurements using the Fluorstar Omega (Fluorstar Omega, BMG Labtech, Ortenberg, Germany) with excitation/emission wavelengths of 560 nm (A560) and 590 nm (A590), respectively. All procedures were performed in triplicate and on three separate occasions (n = 9).

#### 2.6.3. Qualitative and Quantitative Analysis of Cell Viability Using CFM

For this assay, *C. albicans* biofilm was formed on the specimens as previously described. The biofilms were washed twice with 1000 µL of sterile PBS to remove non-adherent cells. Then, 600 µL of the dye solution from the LIVE/DEAD^TM^ FungaLight^TM^ Yeast Viability kit (TermoFisher Scientific, Marietta, OH, USA), containing SYTO-9 and propidium iodide (IP) [35] was added to each well. The plates were incubated for 30 min in the dark. Excitation/emission was approximately 480/500 nm for SYTO-9 and 490/635 nm for PI. Images were obtained using a confocal fluorescence microscope (LSM 800 with Airyscan, Carl Zeiss, Jena, Germany), with live cells stained green and non-viable cells stained red (qualitative analysis). For quantitative analysis, the fluorescence intensity emitted by the samples and the thickness of the biofilms were evaluated using the Zen Blue software (Version 2.3, Carl Zeiss, Jena, Germany). Three specimens were used per experimental group (n = 3).

### 2.7. Statistical Analysis

The results of surface roughness, colony forming unit count (CFU/mL) and cellular metabolic activity showed non-normal distribution. Therefore, the Mann-Whitney U test was used. The different simulated brushing times (T1, T2, T5) were compared with the non-brushed group (T0). On the other hand, the quantitative data from CFM showed normal distribution and was subjected to the 3-way ANOVA, followed by Sidak’s post-test for multiple comparisons and independent samples. All analyses were performed using IBM^®^ SPSS^®^ Statistics (version 29.0.0.0 , IBM Corporation, Chicago, IL, USA), with a significance level of 5%. Figures were created using GraphPad Prism version 10.5.0 (GraphPad Software, San Diego, CA, USA).

## 3. Results

### 3.1. Surface Roughness

**Figure 1:**
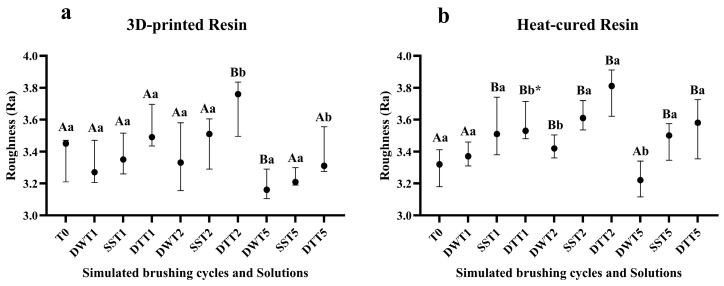
(**a**) Median values and interquartile ranges of roughness for the 3D-printed resin, according to different cleaning/disinfection agents and simulated brushing periods. (**b**) Median values and interquartile ranges of roughness for the heat-cured resin, according to different cleaning/disinfection agents and simulated brushing periods. Different capital letters indicate significant differences in roughness values at T1, T2 and T5 when compared to T0 (*p* < 0.05), while different lowercase letters indicate significant differences between cleaning solutions within the same brushing time (*p* < 0.05), according to the Mann–Whitney U test. * Significant difference observed only between the distilled water and dentifrice groups. **Captions**: **T0**: No brushing; **DWT1**: 1 year of brushing with distilled water; **SST1**: 1 year of brushing with disinfectant liquid soap solution; **DTT1**: 1 year of brushing with dentifrice; **DWT2**: 2 years of brushing with distilled water; **SST2**: 2 years of brushing with disinfectant liquid soap solution; **DTT2**: 2 years of brushing with dentifrice. **DWT5**: 5 years of brushing with distilled water; **SST5**: 5 years of brushing with disinfectant liquid soap solution; **DTT5**: 5 years of brushing with dentifrice.

For the printed resin, the highest surface roughness values were observed after simulated brushing for two years with dentifrice (T2) (Figure 1a). For the other solutions, there were no significant changes in roughness values at most time points. For the heat-cured resin, an increase in surface roughness was noted from T1 onward after brushing with dentifrice and disinfectant liquid soap (Figure 1b). The mean and standard deviation values are presented in Table S1. In addition, for both resins, a reduction in roughness was observed at T5.

### 3.2. Assessment of Cell Proliferation by Counting Colony Forming Units (CFU/mL)

**Figure 2:**
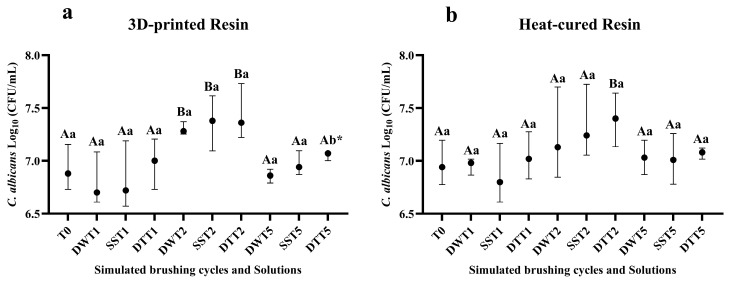
(**a**) Median values and interquartile ranges of Log-transformed *C. albicans* CFU/mL for the 3D-printed resin, according to different cleaning/disinfection agents and simulated brushing periods. (**b**) Median values and interquartile ranges of Log-transformed *C. albicans* CFU/mL for the heat-cured resin, according to different cleaning/disinfection agents and simulated brushing periods. Different capital letters indicate significant differences in CFU/mL at T1, T2 and T5 when compared to T0 (*p* < 0.05), while different lowercase letters indicate significant differences between cleaning solutions within the same brushing time (*p* < 0.05), according to the Mann–Whitney U test. * Significant difference observed only between the distilled water and dentifrice groups. **Captions**: See Figure 1.

Two years of simulated brushing, regardless of the solution used, increased *C. albicans* cell proliferation on the printed resin samples (Figure 2a). On the other hand, after five years of brushing (T5), a reduction in CFU/mL counts was observed across all groups. Regarding the het-cured resin, an increase in *C. albicans* biofilm formation was also observed in the T2 for dentifrice group (Figure 2b). The mean and standard deviation values are presented in Table S2.

### 3.3. Assessment of Cellular Metabolic Activity Using the alamarBlue^®^ Assay

**Figure 3:**
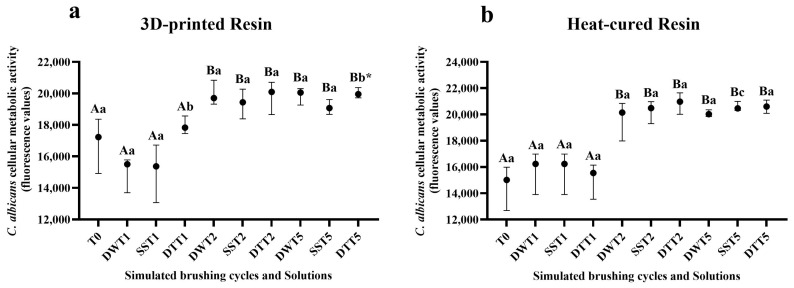
(**a**) Median values and interquartile ranges of *C. albicans* cellular metabolic activity (alamarBlue assay, fluorescence values), according to different cleaning/disinfection agents and simulated brushing periods of the 3D-printed resin. (**b**) Median values and interquartile ranges of *C. albicans* cellular metabolic activity (alamarBlue assay, fluorescence values) according to different cleaning/disinfection agents and simulated brushing period of the heat-cured resin. Different capital letters indicate significant differences in fluorescence values at T1, T2 and T5 when compared to T0 (*p* < 0.05), while different lowercase letters indicate significant differences between cleaning solutions within the same brushing time (*p* < 0.05), according to the Mann–Whitney U test. * Significant difference observed only between the disinfectant liquid soap solution and dentifrice groups. **Captions**: See Figure 1.

Table S3 displays the mean and standard deviation values of fluorescence related to cellular metabolic activity, as measured by the alamarBlue^®^ assay. For both resins (3D-printed and heat-cured), a significant increase in fluorescence values was observed after two years of simulated brushing across all solutions, corroborating the CFU/mL data for the 3D-printed samples. A similar pattern of metabolic activity was observed after five years of simulated brushing (Figure 3a,b).

### 3.4. Qualitative and Quantitative Analysis of Cell Viability Using CFM

**Figure 4:**
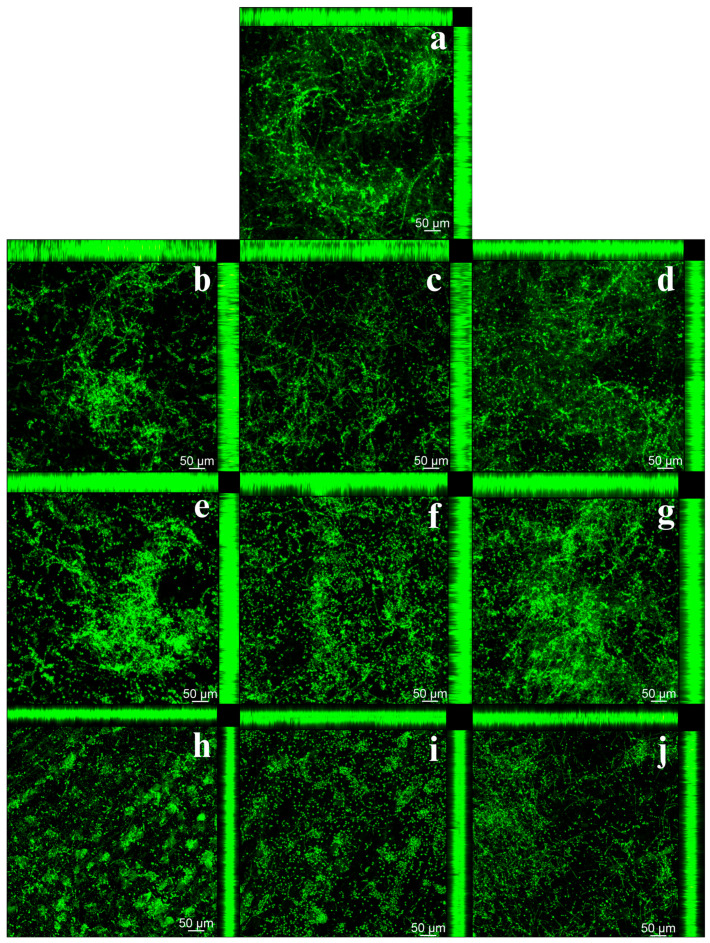
Confocal Fluorescence Microscopy (CFM) images of *C. albicans* biofilms on 3D-printed resin samples before and after simulated brushing with the cleaning/disinfection agents. (**a**) Biofilm of the control group (T0—No brushing); (**b**) Biofilm after 1 year of brushing—Distilled water; (**c**) Biofilm after 1 year of brushing—Soap solution; (**d**) Biofilm after 1 year of brushing—Dentifrice; (**e**) Biofilm after 2 years of brushing—Distilled water; (**f**) Biofilm after 2 years of brushing—Soap solution; (**g**) Biofilm after 2 years of brushing—Dentifrice; (**h**) Biofilm after 5 years of brushing—Distilled water; (**i**) Biofilm after 5 years of brushing—Soap solution; (**j**) Biofilm after 5 years of brushing—Dentifrice.

**Figure 5:**
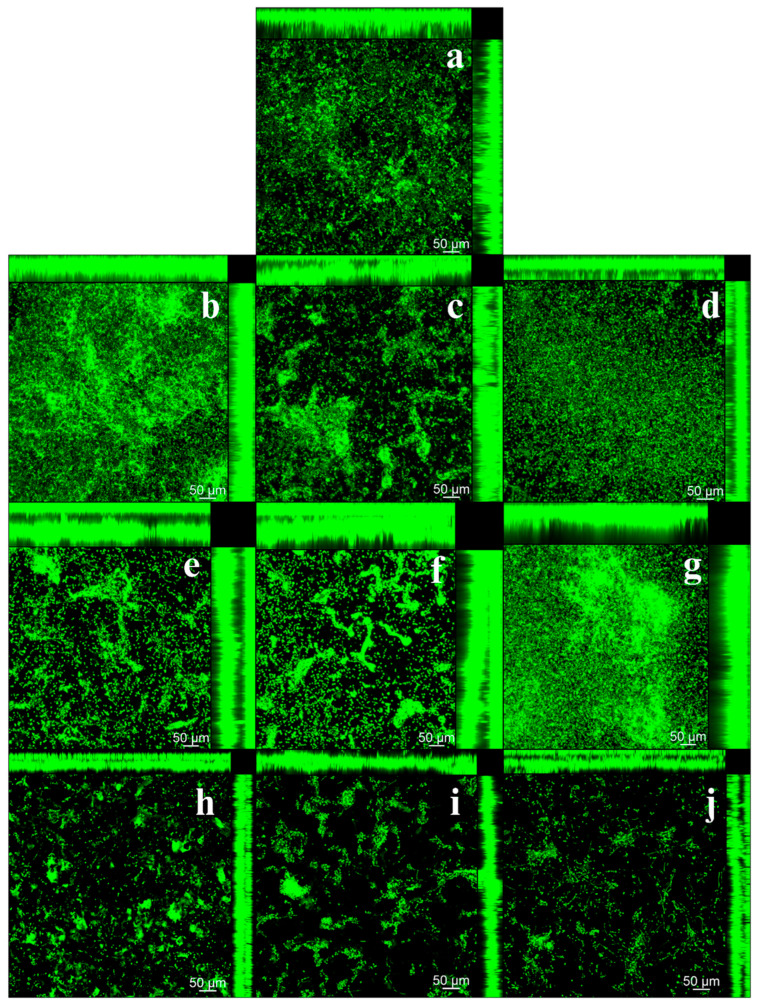
Confocal Fluorescence Microscopy (CFM) images of *C. albicans* biofilms on heat-cured resin samples before and after simulated brushing with the cleaning/disinfection agents. (**a**) Biofilm of the control group (T0—No brushing); (**b**) Biofilm after 1 year of brushing—Distilled water; (**c**) Biofilm after 1 year of brushing—Soap solution; (**d**) Biofilm after 1 year of brushing—Dentifrice; (**e**) Biofilm after 2 years of brushing—Distilled water; (**f**) Biofilm after 2 years of brushing—Soap solution; (**g**) Biofilm after 2 years of brushing—Dentifrice; (**h**) Biofilm after 5 years of brushing—Distilled water; (**i**) Biofilm after 5 years of brushing—Soap solution; (**j**) Biofilm after 5 years of brushing—Dentifrice.

In the qualitative analysis of *C. albicans* biofilm, using CFM, an increase in the number of viable cells was observed on the 3D-printed resin samples in the 2-year simulated brushing groups, regardless of the solution used, in agreement with the other results (Figure 4e–g). Additionally, a decrease in fluorescence was noted after five years of simulated brushing across all groups (Figure 4h–j). In relation to heat-cured resin, there was an increase in biofilm formation after two years of simulated brushing, especially after brushing with dentifrice (Figure 5e–g). However, after 5 years, a reduction in biofilm was observed, evidenced by the lower number of live cells, regardless of the solution used (Figure 5h–j). These findings agree with the results obtained in the CFU/mL count. In addition, comparison between the two resins revealed a higher proliferation of *C. albicans* in the heat-cured resin samples.

**Figure 6:**
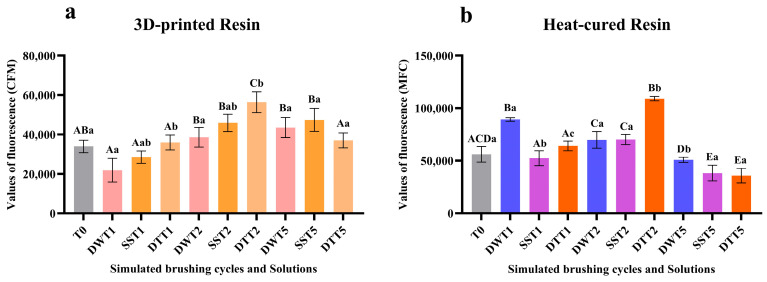
(**a**) Mean and standard deviation of fluorescence values obtained by CFM for the 3D-printed resin, according to different cleaning/disinfection agents and simulated brushing periods. (**b**) Mean and standard deviation of fluorescence values obtained by CFM for the heat-cured resin, according to different cleaning/disinfection agents and simulated brushing periods. Different capital letters indicate significant differences in fluorescence values between columns (*p* < 0.05), while different lowercase letters indicate significant differences between cleaning/disinfection agents within the same brushing period (*p* < 0.05), according to three-way ANOVA and Sidak’s post hoc test. **Captions**: See Figure 1.

Table S4 presents the results of the three-way ANOVA for the fluorescence values of the *Candida albicans* biofilm analyzed by confocal fluorescence microscopy, considering the interactions between the tested factors (Resin × Solution × Time). Figure 6 presents the quantitative analysis of the images obtained via CFM, based on fluorescence intensity values. In general, for both resins, there was an increase in fluorescence values after simulated brushing for 2 years (T2) in all solutions, with greater evidence for the groups brushed with disinfectant soap and dentifrice, which showed a significant difference in relation to distilled water group. After five years (T5) of simulated brushing, a decrease in fluorescence values was observed for most groups, except for the printed resin samples immersed in distilled water and soap.

**Figure 7:**
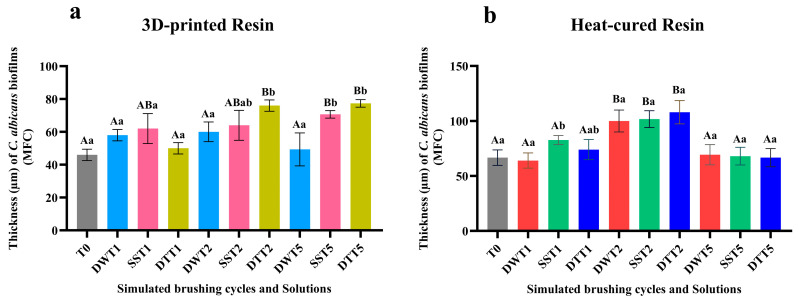
(**a**) Mean and standard deviation of *C. albicans* biofilm thickness (µm) obtained by CFM for the 3D-printed resin, according to different cleaning/disinfection agents and simulated brushing periods. (**b**) Mean and standard deviation of *C. albicans* biofilm thickness (µm) obtained by CFM for the heat-cured resin, according to different cleaning/disinfection agents and simulated brushing periods. Different capital letters indicate significant differences in biofilm thickness between columns (*p* < 0.05), while different lowercase letters indicate significant differences between cleaning/disinfection agents within the same brushing period (*p* < 0.05), according to three-way ANOVA and Sidak’s post hoc test. **Captions**: See Figure 1.

The biofilm thickness values presented in Figure 7 were obtained through analysis of images captured by CFM, and the three-way ANOVA results for the biofilm thickness values, considering the interactions among the tested factors (Resin × Solution × Time), are provided in Table S5. For both resin types, an increase in biofilm thickness was observed after two years of simulated brushing (T2), except for the conventional resin samples brushed with distilled water. However, after five years of simulated brushing (T5), biofilms formed on heat-cured resin samples displayed decreased thickness (Figure 7b). These results agree with the fluorescence intensity values obtained by CFM.

## 4. Discussion

Additive manufacturing has been applied in the production of prosthetics to optimize and streamline the fabrication process. There are notable differences between conventional resins (heat-cured) and those produced through 3D printing, particularly regarding their physical and mechanical properties and biofilm formation. Furthermore, studies show that these properties can be altered by cleaning methods, such as brushing [23]. Thus, the objective of this in vitro study was to evaluate the effects of simulated mechanical brushing (1, 2, and 5 years) with cleaning and disinfection agents on the surface roughness and biofilm formation on denture base resins obtained by the conventional method and by 3D printing. The null hypotheses were rejected, since brushing altered the roughness and biofilm formation of *C. albicans* on the samples of both resins.

The results of the present study showed that, regardless of the type of resin, surface roughness increased after two years of simulated toothbrushing with dentifrice. Similar findings were reported by Chang et al. (2021) [36], who observed increased roughness in both conventional and CAD/CAM-processed materials following simulated brushing with a toothbrush and dentifrice. In contrast, a study using the same types of materials found no increase in roughness after a simulated four-year period. However, in that case, the brushing substance was a mixture of water and ground alkali soap, suggesting that the use of dentifrices, depending on their abrasiveness, may lead to greater wear on denture bases [37].

The increase in surface roughness after brushing observed in the present study is probably due to the abrasive substances found in toothpastes. Calcium carbonate (CaCO_3_) is a mineral with abrasive properties arranged heterogeneously, with irregular shape and size [38,39]. CaCO_3_ is typically found in several brands of commercially available dentifrice. However, the dentifrice used in the present study contains hydrated silica (SiO_2_·nH_2_O), a mineral with greater hardness than CaCO_3_ [40]. It has been observed that abrasion values can increase by up to tenfold when using dentifrices containing hydrated silica in combination with toothbrushes, compared to brushing with water alone [41]. Therefore, it is essential to assess the abrasiveness levels of dentifrices, as they have a direct impact on the surface roughness, hardness, and optical properties of polymeric materials following brushing [42].

In the samples made from heat cured resin, an increase in surface roughness was also observed after brushing with a disinfectant liquid soap (Lifebuoy^®^). To date, no studies have been identified in the literature that have used Lifebuoy soap in prolonged brushing protocols. However, a previous study concluded that brushing for 10 cycles with the same solution did not alter the rough surface of a heat-cured denture base acrylic resin [23]. Another study assessed the effects of brushing for 10 s combined with immersion in disinfectant solutions on both conventional and 3D-printed resins and an increase in roughness was observed in the conventional resin, regardless of the solution used [43]. It is important to note that both studies employed short-term brushing protocols (10 s), which limit direct comparisons with the findings of the present study.

Although roughness increased after 2 years of brushing (T2), at T5, for both resins, a reduction in roughness was observed. This reduction, caused by prolonged brushing, was probably due to the repetitive abrasive contact of the brush bristles, combined with cleaning and disinfecting agents, which progressively removed the surface irregularities of the resin and produced a polishing effect.

In general, following simulated brushing, heat-cured resin samples exhibited higher surface roughness compared to those produced by 3D printing. This difference in roughness values can be attributed to intrinsic material characteristics and processing methods, both of which may contribute to increased wear during brushing. In the case of heat-cured resins, the manual mixing of powder and liquid can incorporate air bubbles, which may lead to higher surface roughness [44]. Additionally, during and after the polymerization process, the release of residual monomers can further contribute to surface roughness [5]. Such factors are not present in the 3D printing process, which contributes to the smoother surface of the material. Additive manufacturing offers a standardized workflow, reduces fabrication time, and minimizes the occurrence of defects compared to conventional methods. Furthermore, the roughness of printed resins is influenced by the printing orientation, commonly set at 0°, 45°, or 90°, with the 90° orientation generally resulting in lower roughness compared to other angles [27].

Biofilm formation on denture bases may be associated with surface characteristics of the resins, particularly increased roughness [45,46]. In the present study, it can be inferred that the increased surface roughness contributed to biofilm development, as higher deposition of *C. albicans* was observed on rougher surfaces [47]. The results demonstrated that simulated brushing over a two-years period, for both resin types and most tested solutions, especially those involving dentifrice, led to increased cell proliferation and metabolic activity of the *C. albicans* biofilm. Additionally, confocal microscopy images supported these findings by revealing a greater concentration of microorganisms, particularly after two years of brushing.

For some groups, the surface roughness of both heat-cured and 3D-printed resin samples decreased after five years of simulated brushing (T5). This reduction may be attributed to the progressive wear of more prominent surface irregularities, resulting in the attenuation of grooves and a more leveled surface topography. Additionally, at this point (T5), a reduction in *C. albicans* cell number was observed, as indicated by colony-forming unit counts and CFM images (fluorescence values and biofilm thickness). However, metabolic activity (alamarBlue^®^) remained elevated after five years of simulated brushing. This outcome may be explained by the mechanism of the alamarBlue assay, which relies on the penetration of oxidized resazurin into cells. There are some potential disadvantages that should be considered regarding this test. AlamarBlue is not a direct cell counting technique and the fluorescence or absorbance signal may be affected by changes in both the number of living cells and cellular metabolism. In this way, alamarBlue detects the metabolic activity of cells, not necessarily their ability to replicate. Metabolically active but non-viable cells (e.g., damaged or stressed) can still reduce the reagent, generating false positives regarding viability [48]. In addition, the lack of a strong correlation between CFU counts and metabolic activity may be attributed to cell aggregation or hyphal formation, which can reduce CFU measurements while leaving metabolic activity unaffected [49].

Different denture fabrication methods can influence microbial adhesion [8]. In the present study, qualitative and quantitative analyses based on CFM images revealed greater biofilm adhesion and thickness on samples produced using the conventional heat-cured method compared to those fabricated by 3D printing. As previously reported, material-inherent characteristics and processing techniques influence the surface properties of denture base resins and, consequently, affect biofilm adhesion and formation. Although roughness was initially standardized, other surface properties, such as wettability, may have contributed to these results. The findings of this study are consistent with a recently published literature review in which the authors systematically summarized available in vitro evidence regarding the adhesion potential of *C. albicans* on digitally fabricated acrylic resin samples compared to conventionally processed ones [50]. The meta-analysis indicated that biofilm coverage, as measured by CFU/mL counts or optical density values, is lower on digitally fabricated resin samples than on conventionally fabricated ones. This suggests that digitally manufactured materials provide a less favorable environment for *C. albicans* colonization.

Although the etiology of denture stomatitis is considered multifactorial, the adhesion of microorganisms, mainly Candida species, to the inner surface of the denture bases and the formation of biofilm are considered primary factors in the onset of this disease [51,52,53,54,55]. Therefore, considering the findings of the present study, non-abrasive solutions and soft brushes should be recommended for cleaning dentures, thus avoiding greater accumulation of biofilm over time.

The present study has some limitations. For instance, it is challenging to compare simulated brushing with the actual brushing patterns of denture wearers due to variables such as applied force and brushing movements. In addition, the linear brushing applied to the samples may result in grooves that may not be accurately detected by linear profilometer scans (Ra). Nevertheless, the use of a brushing machine is widely regarded as a simple, cost-effective, and reliable method, with consistency reported between clinical and laboratory studies [56]. Moreover, thermocycling tests were not performed to simulate temperature fluctuations in the oral cavity. Therefore, further studies are needed to establish effective hygiene protocols for both conventionally manufactured and 3D-printed removable dentures, with the goal of preventing oral conditions such as denture stomatitis.

## 5. Conclusions

Within the limitations of the present study, it can be concluded that simulated brushing, especially when performed with dentifrice, led to an increase in surface roughness in both resins, with more pronounced effects observed after two years. Additionally, these conditions promoted increased cell proliferation and metabolic activity of *C. albicans*. The qualitative and quantitative analyses of the CFM images further support these findings.

## Data Availability

The raw data supporting the conclusions of this article will be made available by the authors on request.

## Supplementary Materials

The following supporting information can be downloaded at: https://www.mdpi.com/article/10.3390/jof11090668/s1. Table S1. Mean and standard deviation values of surface roughness for the resin types, cleaning/disinfection agents, and simulated brushing time. Table S2. Mean and standard deviation (log-transformed) of *C. albicans* CFU/mL values according to resin type, cleaning/disinfection agents, and simulated brushing time. Table S3. Mean and standard deviation values of *C. albicans* cellular metabolic activity (fluorescence), according to resin type, cleaning/disinfection agents, and simulated brushing time. Table S4. Three-way ANOVA for fluorescence values of *C. albicans* biofilm assessed by confocal fluorescence microscopy. Table S5. Three-way ANOVA for thickness values (μm) of *C. albicans* biofilm assessed by confocal fluorescence microscopy.
