# Supplementary material for: Surface Changes Induced by Brushing Increase Candida albicans Biofilms on 3D-Printed Denture Base Resin
Source: J Fungi (Basel). 2025 Sep 12;11(9):668. doi: 10.3390/jof11090668 (PMC12470312; doi:10.3390/jof11090668)
Supplement: Supplementary file 1 [file jof-11-00668-s001.zip › jof-3810562-supplementary.pdf]

SUPPLEMENTARY MATERIAL

Table S1. Mean and standard deviation values of surface roughness for the resin types, cleaning/disinfection agents, and simulated brushing time.

Resin	Solution	T0	T1	T2	T5
	Distilled water	3.38 ± 0.17	3.32 ± 0.15	3.38 ± 0.27	3.19 ± 0.11
**3D printed**	Soap solution	3.38 ± 0.17	3.44 ± 0.24	3.46 ± 0.16	3.23 ± 0.11
	Dentifrice	3.38 ± 0.17	3.53 ± 0.33	3.70 ± 0.26	3.40 ± 0.20
	Distilled water	3.30 ± 0.13	3.38 ± 0.09	3.43 ± 0.08	3.23 ± 0.14
**Heat-cured**	Soap solution	3.30 ± 0.13	3.56 ± 0.20	3.64 ± 0.13	3.48 ± 0.18
	Dentifrice	3.30 ± 0.13	3.58 ± 0.16	3.76 ± 0.16	3.54 ± 0.21

T0: No brushing; T1: 1 year of simulated brushing (10,000 cycles); T2: 2 years of simulated brushing (20,000 cycles); T5: 5 years of simulated brushing (50,000 cycles).

Table S2. Mean and standard deviation (log-transformed) of *C. albicans* CFU/mL values according to resin type, cleaning/disinfection agents, and simulated brushing time.

Resin	Solution	T0	T1	T2	T5
	Distilled water	6.86 ± 0.32	6.81 ± 0.29	7.34 ± 0.17	6.87 ± 0.12
**3D printed**	Soap solution	6.86 ± 0.32	6.88 ± 0.41	7.36 ± 0.25	6.97 ± 0.13
	Dentifrice	6.86 ± 0.32	6.97 ± 0.26	7.32 ± 0.41	7.06 ± 0.11
	Distilled water	6.97 ± 0.21	6.96 ± 0.12	7.20 ± 0.48	7.03 ± 0.18
**Heat-cured**	Soap solution	6.97 ± 0.21	6.88 ± 0.33	7.33 ± 0.40	7.05 ± 0.31
	Dentifrice	6.97 ± 0.21	7.05 ± 0.30	7.42 ± 0.30	7.06 ± 0.08

T0: No brushing; T1: 1 year of simulated brushing (10,000 cycles); T2: 2 years of simulated brushing (20,000 cycles); T5: 5 years of simulated brushing (50,000 cycles).

Table S3. Mean and standard deviation values of *C. albicans* cellular metabolic activity (fluorescence), according to resin type, cleaning/disinfection agents, and simulated brushing time.

Resin	Solution	T0	T1	T2	T5
	Distilled water	16,728 ± 1,851	15,819 ± 2,817	19,864 ± 1,062	19,852 ± 872
**3D printed**	Soap solution	16,728 ± 1,851	15,064 ± 1,927	19,374 ± 1,184	19,222 ± 834
	Dentifrice	16,728 ± 1,851	17,974 ± 606	19,737 ± 1,287	20,182 ± 726
	Distilled water	14,687 ± 2,174	15,477 ± 2,135	19,660 ± 1,413	20,293 ± 857
**Heat-cured**	Soap solution	14,687 ± 2,174	14,870 ± 1,555	20,106 ± 1,340	20,719 ± 655
	Dentifrice	14,687 ± 2,174	15,081 ± 1,321	20,837 ± 949	20,522 ± 617

T0: No brushing; T1: 1 year of simulated brushing (10,000 cycles); T2: 2 years of simulated brushing (20,000 cycles); T5: 5 years of simulated brushing (50,000 cycles).

Table S4. Three-way ANOVA for fluorescence values of *C. albicans* biofilm assessed by confocal fluorescence microscopy.

	SS	df	MS	F	*P*
Resin	8405705010	1	8405105010	3088,030	**<.000**
Solution	776450595,6	2	388225297,8	14,227	**<.000**
Time	4985027812	2	2492513906	91,339	**<.000**
Resin * Solution	1141228724	2	570614362,2	20,910	**<.000**
Resin * Time	4595625577	2	2297812788	84,204	**<.000**
Solution * Time	3145640685	4	786410171,2	28,818	**<.000**
Resin * Solution * Time	1454612202	4	363653050,6	13,326	**<.000**
Error	1091543241	40	27288581,02		

In bold, statistically significant values considering p<0.05
Degrees of Freedom (df); Sum of Squares (SS); Mean Square (MS)

Table S5. Three-way ANOVA for thickness values (µm) of *C. albicans* biofilm assessed by confocal fluorescence microscopy.

	SS	df	MS	F	*P*
Resin	4648,167	1	4648,167	88,396	**<.000**
Solution	830,037	2	415,019	7,893	**<.001**
Time	4335,148	2	2167,574	41,222	**<.000**
Resin * Solution	106,778	2	53,389	1,015	0,371
Resin * Time	2671,000	2	1335,500	25,398	**<.000**
Solution * Time	649,630	4	162,407	3,089	**0,026**
Resin * Solution * Time	978,222	4	244,556	4,651	**0,004**
Error	2103,333	40	52,583		

In bold, statistically significant values considering p<0.05
Degrees of Freedom (df); Sum of Squares (SS); Mean Square (MS)
